# A Survey of Basic Daily Living Assistance in Dependency Units during the Morning First Period at Nursing Homes with a Healthcare Dysfunction

**DOI:** 10.3390/nursrep12010013

**Published:** 2022-02-14

**Authors:** José Antonio Camacho-Conde, David Juan Muñoz-Arbona

**Affiliations:** 1 Department of Evolutionary and Educational Psychology, University of Granada, E-51005 Ceuta, Spain; 2 Teaching Technical Advisor for Inter-institutional Educational Programs, Provincial Office of the Ministry of Education in Ceuta, E-51005 Ceuta, Spain; david.m.arbona@gmail.com

**Keywords:** care record, basic activities of daily living, psychological and functional dependence, elderly, quality of care

## Abstract

(1) Background: It is important to evaluate the attention in the basic activities of daily life in the early hours of the day to evaluate the quality of care and to be able to increase the attention of human resources in case of observing an increase in dependency. The purpose was to improve healthcare quality in nursing homes, correctly identifying the work burden and incidents of daily planning, and completing the work plan by nursing assistant staffing. (2) Methods: The sample is based on 70 elderly people. The analysis used an observational trial every workday over a six-month period. An ad hoc sheet was prepared to collect socio-demographic data on each participant, and the Barthel Index was applied to the study subjects. A daily record of three basic activities of daily living (BADL), such as dressing, bathing, and eating, was kept. (3) Results: Our results showed a significant evolution in both units, but it was in the psychogeriatric unit in which higher compliance with the schedule and higher maintained stability was reached. (4) Conclusions: The use of some BADL registers helped us address situations of imbalance in terms of user assistance and establish an interdisciplinary communication with the nursing team as a way of achieving better organization and compliance with care protocols.

## 1. Introduction

Generalized job dissatisfaction, burnout and frustration in nursing assistants and nurses can lead to problems for patient care [1]. The quality of care in a geriatric home is a constant concern in families with dependent older adults, and family members who perceived that significant improvements were needed in direct care had more negative interactions with other staff [2].

A substantial body of evidence suggests that increased levels of registered nurse (RN) staffing in nursing homes (NHs) are associated with better clinical outcomes [3]. Observational studies and descriptive literature on how RNs spend their time in NHs and acute care settings have used the constructs of direct versus indirect care to categorize and describe the care observed [4].

Direct care refers to nursing and interdisciplinary assessment, physical care, administration of treatments, and psychological care. Indirect care includes documentation (i.e., reading and writing in medical records), supervision, management, and other activities that are performed away from residents but on their behalf to coordinate and manage their care experience and environment [5]. In this sense, geriatric care requires implementation of routine checks that help us to adapt and individualize support for dependent elderly people and prevent significant misalignments in their care and support [6].

Insufficient nursing staff can negatively impact all residents in a nursing home. Numerous studies of NHs reveal a strong positive relationship between the number of nursing home staff who provide direct care to residents on a daily basis and the quality of care and quality of life of residents [7]. Overall, higher nurse staffing improves both the process and outcome measures of nursing home quality [8].

Burnout syndrome is a factor that favors worse geriatric care. There is an important relationship between resistant personality, burnout and health professionals from gerontological centers [9]. Both understaffing and job overload increase burnout [10], and also consider the implications this holds for patient care [11]. Research has also reported burnout levels to be highest amongst those who had organizational issues within the homes in which they worked, such as lack of equipment and personnel, low salaries, workload, administrative mechanisms and bureaucracy [12].

Functional assessment is a multidimensional and often interdisciplinary diagnostic process, which assesses and quantifies an older adult’s medical, psychosocial, and functional status [13]. Information gathered in this process is used by practitioners, patients, and families to develop a comprehensive plan for therapy and future care decisions and can also help in the process of long-term care decision making. Numerous research efforts exist that monitor activities of daily living [14,15,16], these systems usually collect data continuously or when someone is engaged in an activity of interest. Even though many researchers developed systems to monitor ADL activities, they have largely focused on identifying what activity was performed and if the activity was completed or not.

The quality of care cannot be measured in an evolutionary manner without performing data collection addressing several activities of daily living (ADL). By establishing a data register of these activities, healthcare professionals can work in ways that do not only respond to identified needs, but should also be oriented to incorporate people within the definition and adoption of their care plans [17]. They have not yet captured enough information from the activities to assess individuals’ functional abilities to be able to better predict preclinical disability [18].

The healthcare assessment is a basic tool for observing the evolution of a patient and to evaluate whether this evolution is valid and systematic. To ensure good care practices, it is necessary to keep extensive paper or electronic records [19]. This fact allows us to compare the data as a basis for future studies, establishing comparisons between centers and also enables us to make the necessary improvements.

The residential environment requires greater efficiency in its management. It is for this reason that it is necessary to implement standardized care times based on the type of resident through the design of tools that allow care time calculations [20]. The use of previous registers allows us to identify what is to be quantified (time-keeping system) and what type or number of people should be evaluated by analyzing the data to compare its validity and robustness.

Scheduled supervision enables us to record and assess several activities, the average time, and any delays and also help us to identify the causes for each group or unit. The quality of care [21,22,23] in the residence should be based on innovation and continuous improvement [24] through the use of a properly sized professional staff.

The purpose of our study was twofold. The first was to evaluate which basic and instrumental activities of daily living improved in the psychogeriatric units after carrying out a control registry. The second was to consider whether this observational survey could itself become a tool to improve geriatric care by providing rapid data by Personal Digital Assistant (PDA), assessing care and care burdens. This last goal interested us since we started from unstructured care in a timely manner and the baseline could indicate us about the initial performance and the final performance at the end of the study.

## 2. Materials and Methods

In this paper, an alternative approach to the design, implementation, and analysis of process evaluations for geriatric interventions through a functional analysis was proposed.

### 2.1. Study Design

This study used an observational trial on workdays over a six-month period. To evaluate the care data obtained in this study, the PDA was chosen since this device allows efficient data collection in comparison with data that are manually inserted.

Every 30 s a recording interval, which was selected to occur every 5 min, was established. This type of interval is called ‘fixed interval sampling’ for two reasons: (1) it has been shown that random sampling does not show statistical superiority and (2) fixed interval sampling is a method commonly used in research and can also be applied by healthcare professionals who are not nurses [25]. According to the schedule of the users of the unit, standardized data collection lasts between a minimum of 30 min and a maximum of 4 h. After completing the 30 s interval, regardless of whether the care activity was completed by the Registered Nurse Assistant (RNA), the results of the observations are recorded on the PDA. The data collector did not interact with NH staff or residents.

To minimize subject reactivity, subjects were reminded that the focus was only on the frequency of performance of care activities rather than evaluation of their quality. They received detailed instruction about how the PDA worked and how the data collection protocol was intended to decrease intrusiveness and perceived interference with work. Subjects were observed from the hallway from a distance of at least 6 feet. In essence, the subjects become habituated to being observed. Data collector could easily access on the PDA the definition of each care activity in case a reminder was needed.

At the completion of the study, the principal investigator asked subjects to describe their reactions to being observed at work. Although the evidence was anecdotal, they reported that any awkwardness in being observed diminished as more observations were made.

### 2.2. Setting

Recruitment began on the first day of December, with all the residents belonging to the dependency units. The follow-up was carried out during the months of December to May. The data collection was carried out in the two months following the end of the study.

The subjects were observed from the corridor at a distance of at least 2 m. In this way, the subjects became accustomed to being observed. The trained collection personnel could easily access the definition of each care activity on the PDA and could consult the previous data entry of some other activity to check it as a reminder.

At the end of the study, the Principal Investigator asked the workers in the unit to describe their reactions to being observed at work. The workers reported that the initial discomfort due to being observed started to decrease as the presence of the collecting personnel was incorporated and as more observations were progressively made.

### 2.3. Study Site and Participants

The psychogeriatric unit population consists of 44 residents, and the high dependency unit population has 26. The inclusion criteria were: (1) to be between 65 and 90 years; (2a) have a Barthel index equal to or less than 60 (only for the psychogeriatric unit group) or (2b) have a Barthel index equal to or less than 30 (only for the high dependency group). Exclusion criteria were: (1) <65 years.

### 2.4. Data Sources

In order to calculate the number of residents needed in the survey sample, for a simple random sample the following formula is used n_0_ = 70 (1.96·0.5·0.5)/0.02 for a 95% confidence interval.

In this study, we performed a daily follow-up of basic care of an average of 28 residents in the psychogeriatric unit (60% female and 40% male) and an average of 22 residents in the high-dependency unit (70% female and 30% male) by recording care activity from Monday to Friday for six months. Two data collectors (1 psychologist and 1 nurse) were trained using group and individual instructions. The training included the operation of PDAs and sports watches to maintain observation protocols and observation-motive care activities. Data collectors visited the site to familiarize themselves with the physical design before beginning the study.

From the 19th to 30th of November, a biweekly pilot study was initiated in order to observed the general assistance and the number of residents who remained in the units at the time interval between 9:15 and 10:00 a.m.

### 2.5. Measurement

#### 2.5.1. Socio-Demographic Data

An ad hoc sheet was prepared to collect socio-demographic data on each participant (Table 1).

#### 2.5.2. The Barthel Index

The Barthel Index [26] is an ordinal scale that measures functional independence in the domains of personal care and mobility in patients with chronic, disabling conditions, especially in the rehabilitation settings. Two main versions exist: the original 10-item form and expanded 15-item version. The 10-item version is the most used; it includes evaluation of independency in feeding, moving from wheelchair to bed and return, grooming, transferring to and from a toilet, bathing, walking on a level surface, going up and down stairs, dressing, and continence of bowels and bladder. The Barthel index is used to assess disability and to monitor changes in disability over time. The scoring method takes into account whether the person evaluated receives help while doing each task. The scores for each of the items are summed to create a total score, with higher scores indicating higher levels of independency.

Residents receive numerical scores based on whether they require physical assistance to perform the task or can complete it independently. Items are weighted according to the professional judgment of the developers. A resident scoring 0 points would be dependent in all assessed activities of daily living, whereas a score of 100 would reflect independence in these activities. The interpretation suggested by Shah et al. [27] is: (i) 0–20: Total dependency; (ii) 21–60: Severe dependency; (iii) 61–90: Moderate dependence; (iv) 91–99: Low dependency; and (v) 100: Independence. Some authors have proposed reference scores to facilitate interpretation with a cut-off point of 60 (above 60 implies independence). The Barthel Index measures function at the activity level of the International Classification of Functioning, Disability and Health (ICF) [28].

The internal consistency is sufficient and expressed by a Cronbach alpha of 0.84 [29]. The stability of the Barthel index (Stability) was shown by Ganger and his colleagues by estimating the degree of correlation between two index measurements carried out by the same evaluator (test–retest) [30]. The result is 0.89, which shows the good stability of the test. Ganger et al. measured the inter-rater correlation coefficient, greater than 0.95, which shows a similarity of scores on multiple examiners (Equivalence).

The validity of the criteria used (Concurrent Validity) has been demonstrated in several studies. It has been verified by comparing the BI with other assessment tools such as FIM. In 2002, Hsueh et al. showed a good correlation coefficient between the FIM motor subscale and the BI (r = 0.92) [29]. Another study carried out in 2001 also shows a good correlation with the FIM (r = 0.93) as well as a poor correlation coefficient with the Short-Form Health Survey-36 (SF-36) (r = 0.22) [31].

The test administration takes 5 to 10 min. Information is gathered through observation and questioning of those around them or the person themselves. Scoring is facilitated by a definition for each question. The validated version for the Spanish population has been developed by Bernaola-Sagardui [32].

#### 2.5.3. Registration Model

The registry contains three different categories to identify those residents who remain in bed, those who are cleaned, and those who are dressed (Appendix A, Table A1). The residents were classified into the in-bed category when the latter had not been assisted by auxiliary nursing care personnel, into cleaned category when they were handled at both auxiliary and nursing care levels, and in the dressed category when residents were already clothed, but it had not yet been proceeded with the transfer.

Reliability tests during training were performed using a percentage agreement between the data collectors of only the occurrence and non-occurrence of the observed care. Computing only non-occurrence is a reliability method that allows one to understand whether data collectors agree when care did not occur. These reliability computations have been recommended in the research setting by researchers using direct observation and applied behavior analysis as research methods [33]. The tests offered inter-rater reliabilities of 0.87 and 0.95 for occurrences only and no occurrences only, respectively, of observed nursing auxiliary care. During data collection, site reliability tests of 10% of the total observations yielded 0.82 for occurrence only and 0.85 for no occurrence only agreements. Descriptive statistics, including means, counts, and percentages of the categorical variables of the study, were counted.

The Registration Model or Registry Nursing Observation Measure (RNOM) has face validity since its development was based on published clinical guidelines, descriptive literature, research, and RNA regulatory guidelines for surveyors that define recommended RNA care activities. We affirm that we found apparent validity of the measure in reasonable sources [34,35]. On the other hand, we established content validity by having two co-authors review the original lists of RNA care activities derived from the standard-of-care. In addition, we compared the final RNOM that was obtained in order to verify that observable RNA care activities identified in the standards were not excluded.

### 2.6. Ethical Consideration

This study was part of an internal improvement established in the care protocols that does not require informed consent because it is part of the care task to exercise control of the direct care processes. A local ethics committee ruled the formal ethical approval of this study, considering the relevance of the research, the methodological rigor and compliance with the scientific, technical and ethical, national and international standards that govern this type of research. A favorable report was obtained from the Research Ethics Committee of the first Author’s Center (code: 0001-Mont).

### 2.7. Data Analysis

The present retrospective study was completed in 2002. It aimed to encourage geriatric and/or gerontological professionals to implement this type of record because, in almost 15 years, we have not found significant studies providing data on the measurement of basic residential care in the period of greatest burden for geriatric assistants (at the beginning of the day) when they must lift a resident, wash/clean, and then dress them, among other activities.

Statistical analyses were done using the statistical program IBM SPSS Statistics V25.0 for Windows (IBM Corp., Armonk, NY, USA), and presented as descriptive and correlational statistics (sample size, percentage, and Pearson’s correlation). *p*-values < 0.05 were considered statistically significant.

## 3. Results

### 3.1. Participants

The Barthel index for the residents located in the psychogeriatric unit fluctuates from 60 to 0 points (Mean (M) = 43; Standard Deviation (SD) = ±9.7 (at the beginning of the study) M = 34; SD = ±10.5 (at the end of the study), and the Barthel index for the ones who are in the high dependency unit scores ranges from 5 to 0 points, except for one case that reached 30 (M = 5 SD = ±3.7 (at the beginning of the study); M = 3.9 SD = ±2.7 (at the end of the study).

### 3.2. Descriptive Data

The sociodemographic variables (sex, age, marital status and residence before admission) were collected through the center’s database, all under the guidelines dictated by Organic Law 15/1999, of December 13, Protection of Personal Data [36], and controlled by randomization. The most relevant variables are shown in Table 1.

### 3.3. Main Results

According to the pilot study, in the psychogeriatric unit, 43 residents remained bedridden, 11 were cleaned, and five were dressed in their rooms. On the same dates, but on one occasion based on the data collected from the high-dependency units, 112 residents remained in bed, six were cleaned, and 12 were dressed in the room. If we take the month of December as a reference, we perceive a greater mismatch in care because the full month was considered (Table 2 and Table 3).

**Figure 1:**
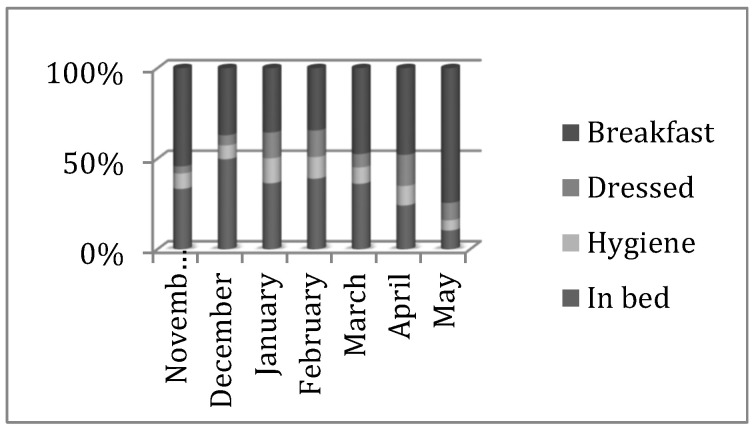
Overall evolution of basic residential care in the Psychogeriatric Unit at the accumulated percentage level.

Our results show a significant evolution in both units, but it was in the psychogeriatric unit in which a higher compliance with the schedule and a higher maintained stability was reached from April onwards. The improvement could be observed in the month of April and decreased until the month of May. Those residents evaluated in the study hour interval who were bedridden moved from 19% to 3% during the month at the end of the study. This indicates an improvement in care assistance, that is to say, 93% of the residents were out of bed by 10 AM, only 1% was being cleaned, and 3% was being dressed and waiting to have breakfast (Figure 1).

**Figure 2:**
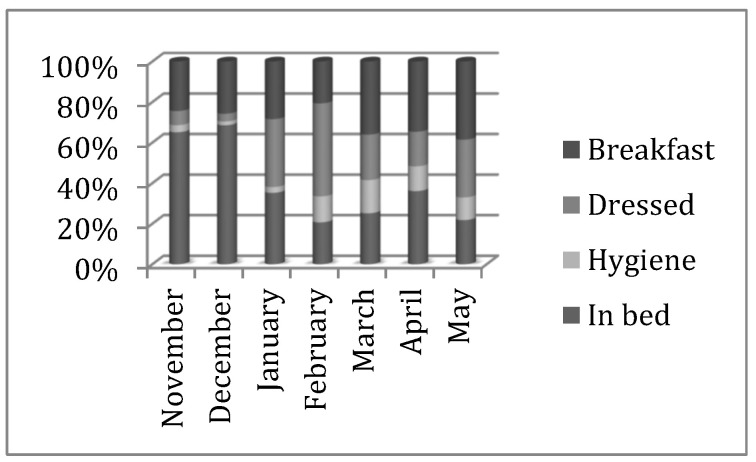
Overall evolution of basic residential care in the High-Dependency Unit at the accumulated percentage level.

In the high dependency unit, a decline in care assistance could be observed in the month of April, but the maladjustment decreased until the month of May. Those residents evaluated in the study hour interval who had been bedridden changed from 48% to 11% during the month of the end of the study. This indicates an improvement in care assistance, that is to say, 89% of the residents were out of bed by 10 AM, only 6% was being cleaned, and 14% was dressed and waiting to have breakfast (Figure 2).

The percentage of bedridden residents decreased from the month of December with an upturn in the month of April. These events leading to this positive evolution were not gradual and can be checked in Appendix A, Table A1 for each month.

In the two graphs shown above, the mismatch of care assistance by residents and per unit can be observed for a fortnight in the pilot study and for a month in the six months under analysis.

**Figure 3:**
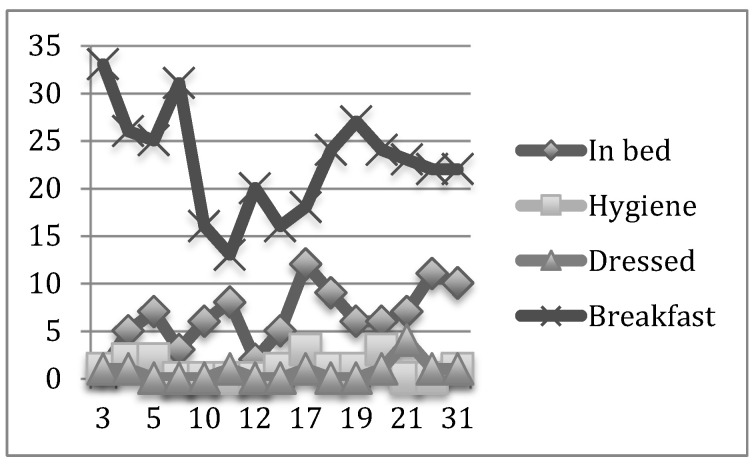
Daily record of the basic activities of daily living (BADL) of the psychogeriatric unit during the initial month.

**Figure 4:**
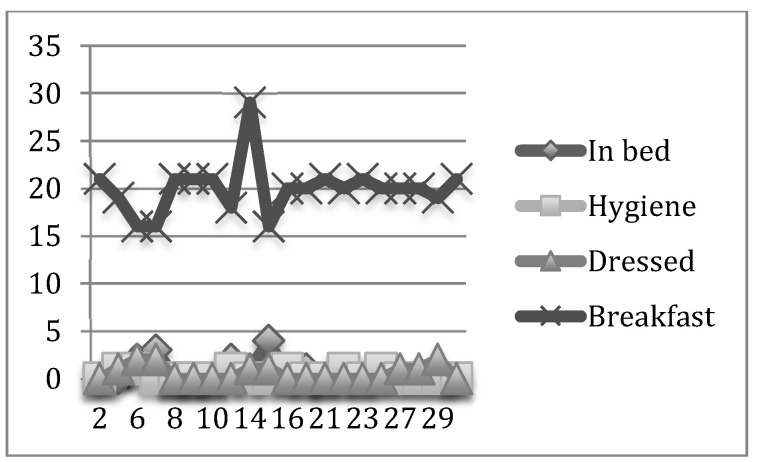
Daily record of the BADL of the Psychogeriatric Unit in the final month.

**Figure 5:**
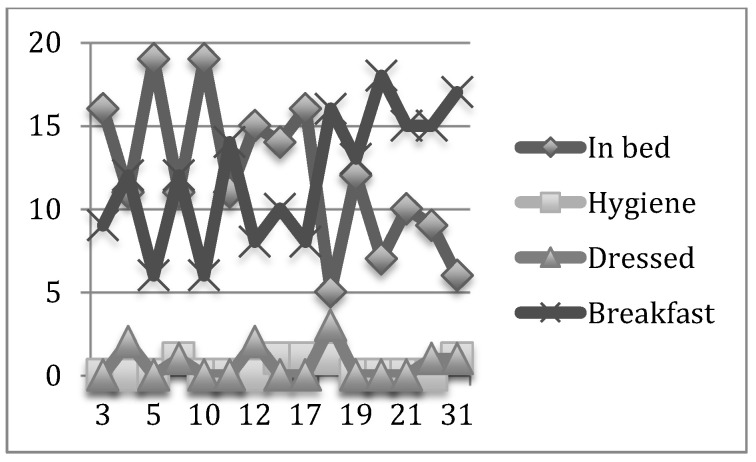
Daily record of the BADL of the High-Dependency Unit in the initial month.

**Figure 6:**
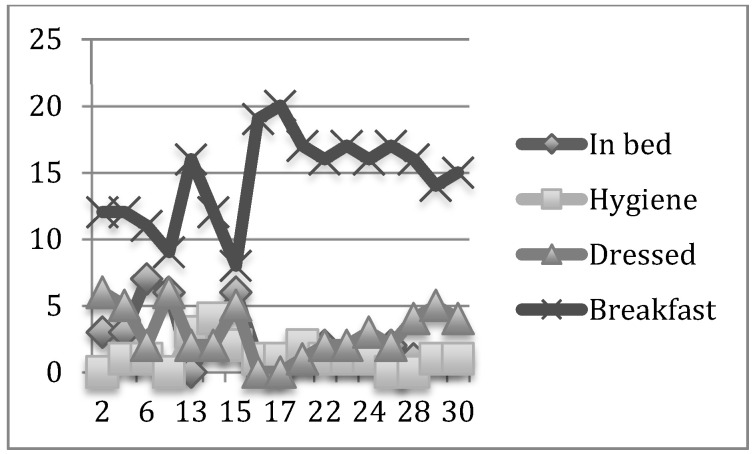
Daily record of the BADL of the High-Dependency Unit in the final month.

In a similar manner, we show the results obtained in the daily record of the initial month (December) and the final month (May) in the psychogeriatric and high-dependency units (Figure 3, Figure 4, Figure 5 and Figure 6, respectively).

The daily average percentage of residents bedridden in the psychogeriatric unit during the study period varied from 6.6% during the month at the beginning of the study to 0.6% during the month at the study end. The daily average percentage of residents cleaned ranged from 1% to 0.3%, and the daily average percentage of residents dressed was 0.7% during both months.

The daily average number of residents in the psychogeriatric unit during the study was 3.63 in bed, 1.01 cleaned, 1.17 dressed, and 26.8 having breakfast.

In contrast, the daily average percentage of bedridden in the high-dependency unit during the study period varied from 12.1% during the month of the beginning of the study to 2.29% during the month at the end of the study. The daily average percentage of residents cleaned ranged from 0.3% to 1.06% and the daily average percentage of residents dressed was between 0.7% and 3%.

The daily average number of residents during the study in the high-dependency unit was 4.45 in bed, 1.18 cleaned, 2.82 dressed, and 13.30 eating breakfast (See Table 2 and Table 3).

## 4. Discussion

### 4.1. Key Results

As it has been possible to expose in the results, there has been a positive evolution and more efficient care since the beginning of the observational study. The differential percentage of bedridden residents was reduced by 6% with respect to the beginning of the observation in the psychogeriatric unit compared to the level of attendance at the end of the study. The differential percentage of residents cleaned was reduced at the end of the study by 0.7% with respect to the level at the beginning of the study and the average daily percentage of residents dressed remained without sufficient variation.

In addition, the differential percentage of bedridden residents was reduced by 9.81% with respect to the beginning of the observation in the high dependency unit compared to the level of care at the end of the study. The daily differential percentage of residents who cleaned experienced an increase of 0.76% with respect to the beginning of the observation and the final level reached in the study, which means a greater investment in this task, helped by the improvement in healthcare activity. Previous and the differential percentage of dressed residents varied by 2.3% between the month of the start of the study and the month of its completion. This has led to a greater efficiency of assistance in this activity by the auxiliary nursing staff.

Our results revealed a more global view of the deficit in basic residential care in the psychogeriatric and high dependency units, although the percentages were similar in both units. We have not found any national report that uses the same measurement criteria as those applied in our study in order to help us to draw comparisons. However, an investigation carried out in the Basque Country and Navarra [20] aimed to assess the need of geriatric assistants in various types of care profiles combining the Barthel index as done in our study and tried to measure the residents’ levels of unmet needs with the objective of designing a common procedure that help us to identify burden of direct care.

On the other hand, at an international level, we can mention the COSMOS study carried out with nursing home patients who have complex problems of physical and mental health, disabilities and social needs, combined with the generalized prescription of psychotropic drugs and has aimed to describe the process of development, content and implementation of the COSMOS trial to observe general aspects of staff care and staff competence, and consequently the quality of life of nursing home patients in general and people with dementia in particular [17].

The daily records of the basic geriatric care units provide a good instrument for quality control in order to handle the geriatric assistance work burden and follow the incidences during the working time [37]. The good evolution in basic geriatric care experienced in both units could have been due to better organization in personnel distribution and in improvement in resident knowledge [38]. Since the high dependency unit is a zone of considerable burden, auxiliary work force reinforcement was incorporated. In this way, the higher ratio of personnel would improve compliance with the established care objectives. In the middle of the same month, the auxiliaries began to work in pairs in the high-dependency unit.

In mid-February, there was an increase in residents in the psychogeriatric unit due to the admission of new people. The lack of personnel was punctual during the months of February and May. It can be viewed in the list of incidents (Appendix A, Table A2) at the end of this report.

The improvement was gradual in both units although it had more fluctuations in the high dependency unit since the residents are generally more dependent on help and therefore, the geriatric assistants face an increase in their workload. From the beginning to the end of the study, there was an improvement of 23% in the breakfast category. This improvement ensured that at least 93% of the residents could be transferred to the dining room for breakfast by 10:00 a.m. In contrast, in the high dependency unit, the variation was 21%. This finding ensured that at least the 69% of the residents could be transferred to the dining room for breakfast by 10:00 a.m. The ones who received food in their rooms were not counted as part of this percentage. A careful analysis of the basic activities of daily living (BADL) we wanted to include, the times assigned in their measurement, and the targeted persons we wanted to examine was necessary for the purpose of our study. In addition, our register was simple and intuitive; this fact allowed us to adapt our register to the real-life situation of each center. Records could be currently obtained on a handheld or personal digital assistant (PDA) computer, which instantly allows computerized data to be viewed and better control of the geriatric assistance and their tasks in the management or central coordination systems of the nursing center to be obtained [39].

We did not go into much detail about the number of geriatric assistants that work in each unit since it is not within the scope of the evaluation of the ratios, but as we have discussed previously, it is true that depending on the increase in resident dependency, the ratio should be modified to maintain an acceptable quality of service [40]. In this sense, during the realization of this study, we realized the need for a geriatric assistant in the high dependency unit from January to look after the residents in a more efficient way. Likewise, the location of geriatric assistants in some sectors in a stable manner without being rotated on a weekly basis allows for better adaptation to the needs of the dependent residents [41,42].

On the other hand, our study has led us to consider that auxiliary nursing care personnel could find themselves overloaded and in need of emotional support. A study prior to this study in the personnel revealed emotional exhaustion and medium-high rates of burnout. This may mean that, whilst burnout can have a profound impact upon the staff that experience it, it also impacts the quality of care given to the patients with whom they work. Moreover, this might mean that staff who are experiencing the negative aspects of burnout. This also may suggest that some aspects of burnout are more influential on care provision than others [12]. In addition, caregiver stress that is associated with low job satisfaction, long hours, low pay, physical demands, shortage of staff and minimal education and training [43,44], and less empathy that may be related to a lower quality of care [45].

### 4.2. Limitations

A primary limitation of this study is its cross-sectional design. A longitudinal approach would have allowed us to better establish a causal relationship between the variables of interest. Furthermore, the lack of studies related to the present study can be added to the limitations of the study. Beyond the fact that the number of clinical samples was small, it should be noted that there was heterogeneity within them, which could have biased the results.

This study has the limitation that it was carried out only in one center, with workers with a mild-moderate level of burnout that would explain the initial data of inefficient care [12,45,46,47]. However, the auxiliary nursing care personnel did not receive any group therapy for their approach, only some personnel reinforcements that have already been exposed in Appendix A, Table A1. It would be important to evaluate it with larger samples and from other centers, even applying some intervention method.

Unfortunately, we did not assess staff turnover but encouraged continuity in data collection at the beginning and end of months 1 (December) and 6 (May). Despite these limitations, this study was able to obtain critical qualitative and quantitative fundamental data to support the progress of the project and allow future inclusion of observational studies in BADL in NHs. Hopefully, the results of this study will demonstrate the efficacy of an observational study on in BADL in NHs.

### 4.3. Interpretation

Work sampling is adequate to measure care activities by RNA. A more refined measurement of the components of “general care” will help to distinguish the activities performed by RNA. Further study is recommended on how to combine qualitative data collection with quantitative data collected through work sampling. Having both types of data will provide a more nuanced description and measurement of how RN care activities are distributed between direct and indirect care [18]. Improved geriatric care supervision would allow for an increase in the quality of the assistance and also allow early morning intake to be at an appointed time, which would facilitate an interval of at least four hours between breakfast and lunch. When a consensus is reached for the evaluation of the results, we will be able to design a computer tool that calculates the estimation of times for a specific user profile [20].

Studies, such as the one we describe in this report, provide a monitoring tool for auxiliary nursing care personnel and facilitate the measure of the different types of tasks that are being carried out in real-time. Performing a daily follow-up of some activities and if possible, measuring the time it takes to complete them, will allow a greater adjustment in the care offered by a residential service to its users and will facilitate the correction of the factors that delay assistance together with the adaptation of the ratios of dependence/independence levels of every resident. This study also provides significant statistical data of direct care with the highest workload time for a geriatric assistant.

The quality of care for elderly residents is a concern that has a clear reference in the United States (US). In the US, the Omnibus Budget Reconciliation Act (1987) marks a before and after point concerning elder care and developed a work system aimed at improving care services to meet the needs of the elderly [48]. This work system, defined as Resident Assessment Instrument (RAI), had a significant impact on the care of the residents in the Western world [49]. In Spain, some attempts were made to implement an RAI with partial results in Galicia [50] and a Resource Utilization Groups version III (RUG III) [11] in other territories. In the Basque Country, only the Zorroaga Residence (Donostia-San Sebastián) has used the RAI system for the past eight years as a method of integral evaluation of its residents [49].

## 5. Conclusions

The study’s findings suggest that it was feasible to measure the frequency of RN direct and indirect care activities using the RNOM and PDA-based data collection protocol. Accurate measurement of RN practice in NHs is important, given both their limited presence and research findings indicating that better clinical outcomes may be associated with increased RN staffing levels [3,51]. Work sampling provides a useful methodology to use for an accurate description of the distribution of RN time between direct and indirect care.

In addition, there is a need for studies to provide empirical evidence to support a greater shift towards evidence-based clinical and organizational practices and work routines for registered nurses. The hope is that registered nurses are trained to practice efficiently and effectively to promote quality care and quality of life in this challenging work environment such NHs.

Nursing directors and nursing home administrators should carefully examine the working conditions of their auxiliary nursing care personnel. More specifically, they should seek to understand the sources of workforce perception stress and be proactive in ways to address it in collaboration with their staff.

The use of RNOM some basic activities of daily living (BADL) registers has helped us to address situations of imbalance in terms of the resident/user assistance. Under the supervision of RNOM, we have been able to establish an interdisciplinary communication with the nursing team as a way of achieving a better organization and compliance with the care protocols.

It is advisable to apply these tools (RNOM + PDA) to all residents because of their usefulness since they are focused on quality of care improvement and the continuous monitoring of the personnel behaviors.

## Figures and Tables

**Table 1:** Sociodemographic data on elderly residents.

	High Dependency		Psychogeriatry	
Indicators	n	%	n	%
Age				
65–80	16	61.54	19	43.18
>80	10	38.46	25	56.82
Sex				
Male	9	34.62	18	40.91
Female	17	65.38	26	59.09
Marital status prior to admission				
Widower	14	53.85	24	54.55
Married	7	26.92	12	27.27
Separated/ Divorced	1	3.85	3	6.82
Single	4	15.38	5	11.36
Current Marital Status				
Widower	17	65.39	27	61.36
Married	4	15.38	10	22.73
Separated/ Divorced	1	3.85	4	9.09
Single	4	15.38	3	6.82
Residence before admission				
Institution	8	30.77	12	27.27
Sons	4	15.38	7	15.91
Spouse	7	26.92	12	27.27
Alone	4	15.38	5	11.36
Other family	1	3.85	7	15.91
Others	2	7.70	1	2.28

**Table 2:** Monthly evolution of the residential basic care in Psychogeriatric Unit.

	B	%	M	H	%	M	D	%	M	Br	%	M	N
November	43	22	4.8	11	6	1.2	5	2	0.6	139	70	15.4	22
December	98	21	6.6	15	3	1	11	3	0.7	340	73	25.7	34
January	82	11	3.9	31	4	1.4	32	5	1.52	589	80	28.1	35
February	85	15	4.7	26	4	1.4	32	6	1.7	425	75	23.2	31
March	64	11	3.7	16	3	0.9	13	2	0.7	488	84	28.7	34
April	45	5	2.3	20	2	1.1	32	4	1.7	758	89	39.9	45
May	13	4	0.6	7	2	0.3	12	3	0.7	309	91	15.4	17
_x_	61		3.63	18		1.01	19		1.17	435		26.8	
σ	32			10			11			201			

B: In bed; H: Hygiene; D: Dressed; Br: Breakfast; M = Average number of residents per day with a basic activities of daily living (BADL).

**Table 3:** Monthly evolution of basic residential care in the High-Dependency Unit.

	B	%	M	H	%	M	D	%	M	Br	%	M	N
November	112	22	12.5	6	6	0.7	12	2	1.3	95	70	10.5	25
December	181	21	12.1	5	3	0.3	10	3	0.7	179	73	11.9	25
January	62	11	3.1	5	4	0.25	59	5	2.95	294	80	14.7	21
February	50	15	2.8	31	4	1.72	111	6	6.16	168	75	9.32	20
March	46	11	2.55	30	3	1.66	41	2	2.27	225	84	12.5	19
April	74	5	3.9	25	2	1.31	35	4	1.84	322	89	16.9	24
May	39	4	2.29	20	2	1.06	51	3	3	247	91	14.5	21
_x_	67		4.45	17		1.18	45		2.82	190		13.3	
σ	52			11			34			86			

B: In bed; H: Hygiene; D: Dressed; Br: Breakfast; M = Average number of residents per day with a basic activities of daily living (BADL).

## Data Availability

The datasets generated during and/or analyzed during the current study are not publicly available because they contain patient identifiable information. Releasing data would breach patient confidentiality.

## Appendix A

**Table A1:** Individual Record Template.

1	L Get Up—Morning Star-Up Activity [9:15–10:00]	Unit: ☐ Psychogeriatric ☐ High Dependency		Day: Register Time: [9:15–10:00]	Resident	Room
Component	Dependency	Task		Time (sg)	Factors	Feedback
		Yes	Not			
In bed	Moderate					
Cleaned	Complete					
Dressed	Complete					
Breakfast	Severe					

Source: Elaborated by the authors, **1:** New residents are assigned to those who have not attended the previous weeks.

**Table A2:** List of incidents that have affected the work pace.

IDATE	INCIDENCE	UNIT	OBSERVATION
**27 November**	A geriatric assistant is incorporated in an hour later to work.	Psychogeriatry	Delay in attendance
**8 January**	Incorporation of an support assistant.	High-Dependency	
**16 January**	Assistants start working in couples.	Both units	Greater flexibility in assignments and compensation charges.
**18 February**	Increased bedridden residents.	Psychogeriatry	It may be that the assistants have begun a new care plan ^1^.
**25 February**	Two assistant missing.	High-Dependency	Delay in attendance. More bedridden residents.
**18 March**	Assistants are located in a residential área stably.	Both units	Promote greater awareness of assigned residents, agility and more personalized attention.
**25 March**	One assistant missing.	Psychogeriatry	Delay in attendance. More bedridden residents.
**8 April**	One assistant missing in the high dependency unit and one replaces of psychogeriatry.	Psychogeriatry	Delay in attendance. More bedridden residents.
**30 April**	Two assistants missing.	High-Dependency	Delay in attendance. More bedridden residents.
**15 May**	One assistant missing.	Psychogeriatry	Delay in attendance. More bedridden residents.

^1^ New residents are assigned to those who have not attended the previous weeks.
